# Analogues of a Cyclic Antimicrobial Peptide with a Flexible Linker Show Promising Activity against *Pseudomonas aeruginosa* and *Staphylococcus aureus*

**DOI:** 10.3390/antibiotics9070366

**Published:** 2020-06-30

**Authors:** Thomas T. Thomsen, Helen C. Mendel, Wafaa Al-Mansour, Alberto Oddo, Anders Løbner-Olesen, Paul R. Hansen

**Affiliations:** 1 Department of Clinical Microbiology, Rigshospitalet, Henrik Harpestrengs Vej 4A, 2100 Copenhagen, Denmark; thomas.thomsen@bio.ku.dk; 2 Department of Biology, Section for functional Genomics, University of Copenhagen, Ole Maaløes Vej 5, 2200 Copenhagen, Denmark; lobner@bio.ku.dk; 3 Department of Drug Design and Pharmacology, Faculty of Health and Medical Sciences, University of Copenhagen, Universitetsparken 2, 2100 Copenhagen, Denmark; hcmendel@gmail.com (H.C.M.); wafaa1992@yahoo.dk (W.A.-M.); albi.oddo@gmail.com (A.O.); 4 Novo Nordisk A/S, Krogshøjvej 44, 2820 Bagsværd, Denmark

**Keywords:** cyclic antimicrobial peptides, antibiotics, multi-drug resistant bacteria

## Abstract

The emergence of multi-drug resistant bacteria is becoming a major health concern. New strategies to combat especially Gram-negative pathogens are urgently needed. Antimicrobial peptides (AMPs) found in all multicellular organisms act as a first line of defense in immunity. In recent years, AMPs have attracted increasing attention as potential antibiotics. Naturally occurring antimicrobial cyclic lipopeptides include colistin and daptomycin, both of which contain a flexible linker. We previously reported a cyclic AMP BSI-9 cyclo(Lys-Nal-Lys-Lys-Bip-O_2_Oc-Nal-Lys-Asn) containing a flexible linker, with a broad spectrum of activity against bacterial strains and low hemolytic activity. In this study, improvement of the antimicrobial activity of BSI-9, against the European Committee on Antimicrobial Susceptibility Testing (EUCAST) strains of *S. aureus*, *E. coli*, *A. baumannii*, and *P. aeruginosa* was examined. This led to synthesis of eighteen peptide analogues of BSI-9, produced in four individual stages, with a different focus in each stage; cyclization point, hydrophobicity, cationic side-chain length, and combinations of the last two. Specifically the modified compound **11**, exhibited improved activity against *Staphylococcus aureus* and *Pseudomonas aeruginosa* with MIC of 4 µg/mL and 8 µg/mL, respectively, compared to the original BSI-9, which had an MIC of 16–32 µg/mL.

## 1. Introduction

The development of antibiotics is one of the greatest accomplishments in modern medicine, treating potentially fatal infections [1]. Nevertheless, the widespread use of antibiotics has led to the selection of resistant isolates, which in turn has resulted in the emergence of multi-drug resistant microorganisms capable of surviving treatment with most or all antibiotics [2]. Of dire importance is the development and prevalence of multidrug resistance in Gram-negative species, such as *Klebsiella pneumonia*, *Acinetobacter baumannii*, and *Eschericia coli*, where pan-resistance is now observed [3]. Therefore, the need for novel antibiotics is urgent, as drug-resistant microorganisms is a global health concern and antibiotic development has declined [4].

Interest in antimicrobial peptides (AMPs) as novel antibiotics, has increased in recent years. Despite their diversity, most AMPs share common features, including a net positive charge, amphipathic character, and short length (<50 amino acids). They have an ancient phylogenetic origin and play an essential role in the innate part of the immune system of multicellular organisms [5]. Their overall cationic charge attracts them to the anionic membrane of the bacterial cell, followed by membrane insertion, thanks to their amphipathic nature. There are various mechanisms which then lead to membrane permeabilization and membrane depolarization, accompanied by membrane disruption or leakage of essential intracellular metabolites, amongst others [6]. Some AMPs kill bacteria by targeting specific intracellular metabolic pathways, thereby disrupting important biological processes [7]. In general, development of resistance to membrane targeting AMPs is considered rare, compared to that of conventional antibiotics. This is likely the result of AMPs killing by disruption of the cellular membrane in a non-target specific way, i.e., the AMPs insert into and disrupt the structural organization of the membrane, as opposed to targeting a single specific target such as an enzyme [8].

The lipopeptide antibiotics colistin (polymyxin E) [9] and daptomycin [10] are currently the last resort drugs used for the treatment of multidrug resistant Gram-negative and Gram-positive bacteria, respectively. They both consist of a cyclic peptide part with a flexible linker. Colistin and daptomycin are highly active against *P. aeruginosa* and *S. aureus*, respectively. However, resistance to these lipopeptides has emerged [11,12], and while arising in highly diverse microorganisms, the resistance mechanisms share overlapping features. Resistance to colistin in Gram-negative species is associated with modification of the lipopolysaccharide found in the outer leaflet of the outer membrane not present in Gram-positive bacteria. Specifically these modifications change the net positive charge (to which polymyxins are attracted) through attachment of phosphoethanolamine (pEtN) or addition of 4-amino-4-deoxy-L-arabinose (L-Ara4N) to LPS [13,14]. Daptomycin resistance, likewise, result in changes to the net negative charge of the Gram-positive membrane, but by changing the content of phosphatidylglycerol (PG) to the more positively charged lysyl-phosphatidylglycerol (L-PG) [15].

Nonetheless, the broad target range, alternative modes of action and evolutionary robustness continue to make AMPs interesting drug candidates and an alternative to the traditional small molecule antibiotics. AMP development is hampered by AMP-specific problems of which in vivo stability, efficacy, and toxicity are the most prominent [16]. Systemic toxicity and stability issues often limit the use of AMPs and most of AMP drugs currently undergoing clinical trials are for topical applications [17]. However, work by Vaara and others, demonstrated how toxicity issues related to polypeptides (polymyxin) might be overcome by turning the molecule into a sensitizing agent, instead of a direct antimicrobial [18]. Thus, current AMP optimization efforts mainly focus on peptidomimetics, for improving stability and antimicrobial activity, while reducing toxicity [19].

**Scheme 1:**
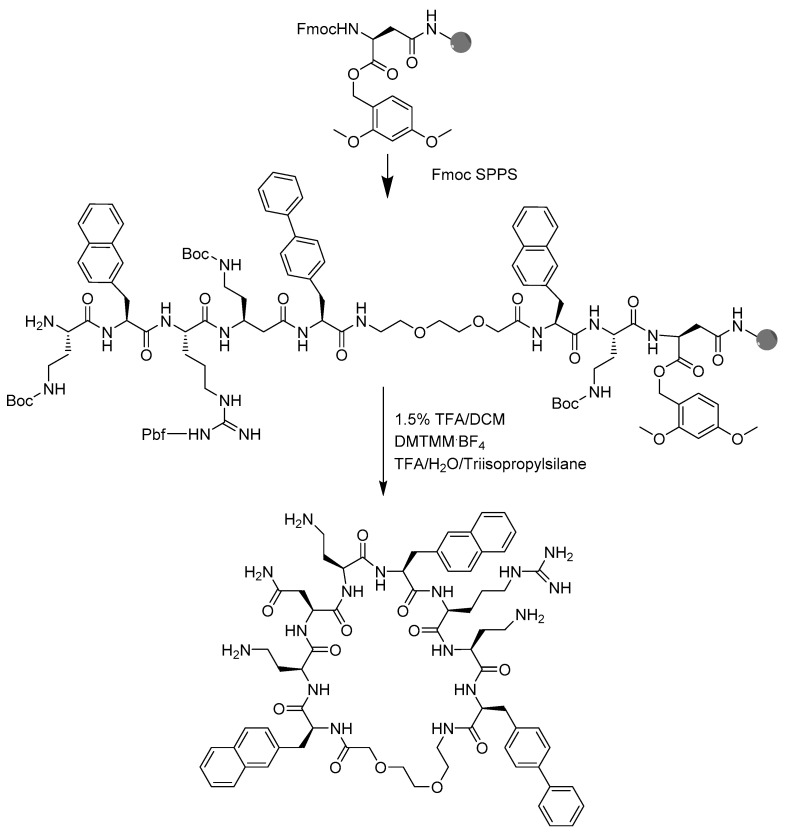
Strategy for solid-phase peptide synthesis of the lead compound **11**.

BSI-9 cyclo(Lys-Nal-Lys-Lys-Bip O_2_Oc-Nal-Lys-Asn) is a cyclic AMP developed by Oddo et al. [20] in a study examining the effects of flexible residues on bioactivity and toxicity in cyclized amphipathic peptides. The flexible residue 8-amino-3,6-dioxaoctanoic acid (O_2_Oc) offers BSI-9, a combination of size, flexibility, and amphipathicity resulting in activity against *E. coli*, *P. aeruginosa*, and drug resistant strains of *A. baumannii* and *S. aureus*, with the minimum inhibitory concentration (MIC) values ranging from 16 µM–64 µM. In addition, BSI-9 displayed low hemolytic activity against red blood cells at 150 µM [20]. In this study, 18 cyclic peptide analogues of BSI-9 were synthesized in four stages (Scheme 1). The cyclic peptides were tested against the bacterial quality control strains set by the European Committee on Antimicrobial Susceptibility Testing (EUCAST); strains *E. coli* ATCC 25922, *S. aureus* ATCC 29213, *A. baumannii* ATCC 19606, and *P. aeruginosa* ATCC 27853.

## 2. Results and Discussion

Charge, overall hydrophobicity, and amphipathicity are important parameters that influence the biological activity and toxicity of AMPs [21]. Studies optimizing linear AMPs often aim to change one or more of these parameters with varying results [22,23,24,25]. Structure–activity studies were also conducted on cyclic peptides [26,27], peptide macrocycles [28], and cyclic peptides, with a flexible linker [29]. In this study, the antimicrobial activity of BSI-9 [20] was improved. We synthesized eighteen peptide analogues of BSI-9 in four stages, focusing on the cyclization point, hydrophobicity, cationic side-chain length, and the combinations of the last two (analogues presented in Table 1).

### 2.1. Stage 1. The Impact of Cyclization Point on Antimicrobial Activity—Replacing Lys with Asn.

The amphipathicity of a peptide is determined by the number of polar and hydrophobic residues, and their position with respect to each other [30]. Therefore, we hypothesized that moving the asparagine—the cyclization point—to different positions in the BSI-9 sequence, would affect bioactivity. In BSI-9, the asparagine (polar, uncharged) was positioned between two lysines (polar, charged). This position was chosen for arbitrary reasons and might not be optimal. Thus, four peptide analogues (**1**–**4**) synthesized in stage 1 (S1) aimed to evaluate the importance of the cyclization point by substituting lysine residues with asparagine at different positions. The MIC and hemolysis results of the S1 peptides are displayed in Table 1. While the peptides displayed varying antimicrobial activity, all four peptides had an equal or higher MIC value compared to BSI-9, against all bacterial strains. We observed a similar level of antimicrobial activity for **1** against *A. baumannii*, compared to BSI-9. In addition, most S1 peptide analogues had a retention time (RT) higher than BSI-9 (Table S1), indicating increased hydrophobicity/amphipathicity. Since stage 1 analogues showed no significant antimicrobial activity, we did not test the hemolytic activity.

### 2.2. Stage 2. The Impact of Manipulating Lipophilicity on Antimicrobial Activity. Replacing Nal (3-(2-naphtyl)-L-alanine) and Bip (L-biphenylananine) with Phe.

Previously, Staubitz et al. reported that reducing the lipophilicity of a peptide by substituting the lipophilic residues could reduce the hemolytic activity, as well as improve antimicrobial activity [22]. Since no significant improvement was observed in stage 1, BSI-9 was used as the lead compound for the synthesis in stage 2 (S2). Here, the (Nal) and (Bip) residues were substituted with the less hydrophobic Phe. Nal and Bip both contain two aromatic rings, while Phe only contains one. Five new analogues of BSI-9 were synthesized (Table 1): compounds **5**–**9**. In peptide **5** (Nal^2^→Phe), **6** (Bip^5^→Phe), and **7** (Nal^7^→Phe) one amino acid was substituted. In compound **8**, two amino acids were substituted (Nal^2,7^, Bip^5^→Phe). The total number of amino acids remained nine. In this stage all peptides had MICs of 32 μM or above against all bacterial strains. The hemolytic activity of all stage 2 analogues was below 10%.

### 2.3. Stage 3. Importance of Amino Acid Sidechain Length on Antimicrobial Activity—Replacing Lys with Dab or Arg.

Stage 3 (S3) investigated the importance of amino acid side chains in relation to antibacterial activity. Specifically, the side chain of the four charged lysine (Lys) residues in BSI-9, were exchanged with charged amino acids, 2,4-diaminobutanoic acid (Dab) and Arginine (Arg). Since Lys and Arg had longer side-chains than, e.g., Dab, these residues were able to insert more deeply into the membrane while the side chain charge was still able to interact with the lipid head groups on the surface; this is known as “snorkeling” [31]. Furthermore, Arg is capable of forming three hydrogen bonds while Lys and Dab can form only two. In compound **9**, we changed all Lys residues to Dab. Peptide **9** inhibited *S. aureus* and *P. aeruginosa* at 8 g/mL and 4 g/mL, respectively, which was an 8-fold increase in activity compared to BSI-9. Although peptide **9** showed an increased hemolytic activity of 76%, compared to BSI-9 (33%), it was decided to use this peptide in the design of additional stage 3 analogues.

Based on the above results, compounds **10**–**13** were designed as a cationic Arg scan of peptide **9**:**10** (Dab^1^->Arg), **11** (Dab^3^->Arg), **12** (Dab^4^->Arg), and **13** (Dab^8^->Arg). In peptide **14,** all Dab residues were replaced with Arg (Dab^1,3,4,8^ ->Arg). In **15** (Dab^1,3^->Arg) were substituted.

The MIC and hemolysis results of the Stage 3 peptides are displayed in Table 1. For peptide **10**, we observed an MIC of 2 µg/mL against *P. aeruginosa* and *S. aureus*. Peptide **11** was still able to inhibit *S. aureus* at 8 μg/mL and *P. aeruginosa* at 4 µg/mL. For both **10** and **11**, the activity against *E. coli* and *A. baumannii* remained unchanged or slightly improved, compared to **9**. Peptide **12** and **13** showed an improvement compared to **9** against *S. aureus*, with an MIC of 1 µg/mL, but not against the other bacterial strains. The MIC of **14** and **15** did not improve, compared to **9**, against any bacterial strains. Peptide **10** showed a lower hemolytic value compared to **9** (from 76% to 66% at 150 μM). Compound **12**, **13**, **14**, and **15** showed hemolytic values of 100%, 80%, 68%, and 100% at 150 µM, respectively. Apart from **13**, which had an RT of 17.9 min, compared to **9** at 17.8 min, the RT times of S3 peptides were similar but lower compared to **9**. They were all higher than BSI-9. 

### 2.4. Stage 4. Reducing Hydrophobicity of Stage 3 Lead Peptide by Replacing Dab with Arg and Bip with Phe. 

Although **12** and **13** were more active against *S. aureus* compared to **11**, they were also significantly more hemolytic (100% and 80%, respectively). Therefore, compound **11** was chosen as the lead compound for stage 4. In stage 4 (S4), we replaced Dab with Arg and Bip of **11** with the less hydrophobic Phe to synthesize three new analogues (**16**–**18**). In **16** (Dab^1,3^->Arg, Bip^5^->Phe) and **17** (Dab^1^->Arg, Bip^5^->Phe) two and three substitutions were done, respectively. In **18**, one substitution was made (Bip^5^->Phe). The MIC of compound 16 did not improve compared to **9**, against *P. aeruginosa* (16 µg/mL) and *S. aureus* (8–64 µg/mL). For all Stage 4 analogues, the activity against *E. coli* and *A. baumannii* remained unchanged or worsened (32 → 64 μg/mL) compared to **11**. (Bip) is often used in structure–activity studies to replace Phe, for example, in the linear antimicrobial peptide Lys-Bip-Lys-Bip-Lys [32], or in the cyclic antimicrobial peptide Polymyxin B [26]. In line with these studies, we observed that the Bip residue was important for antimicrobial activity, which was reduced in the BiP->-Phe analogues (**16**–**18**). 

An indirect correlation between MIC values, % hemolysis, and HPLC retention times (Table S1) was found. In stage 2, MICs were 32 → 64 µg/mL against *S. aureus* and *P. aeruginosa* and we observed a hemolysis below 10%. However, in stage 3, the MICs ranged between 1–32 µg/mL and lysis of red blood cells increased to 36–100%. This might be indicative of a membrane disruption mode of action. The most hemolytic compounds in stage 3 had HPLC retention times between 17.0–17.8 min, while the less hemolytic stage 2 and 4 compounds had values between 13.8–16.0 min. The above correlation was previously observed for both cyclic [33] and linear peptides [34].

Since none of the stage 4 analogues were more active than **11**, this compound remained our best candidate.

## 3. In Vitro Killing Kinetics against *P. aeruginosa* and *S. aureus*

**Figure 1:**
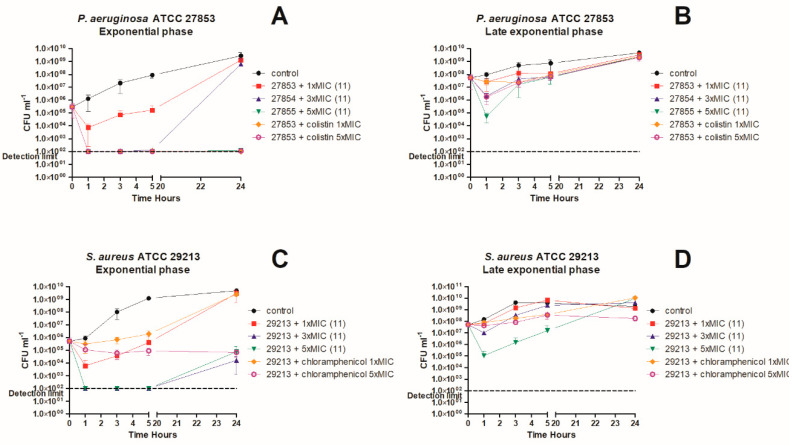
Time-Kill Curves. Bactericidal activities of **11** and colistin at different concentrations against *P. aeruginosa* in the exponential phase (**A**) and late exponential phase (**B**), and chloramphenicol at different concentrations against *S. aureus* in the exponential phase (**C**) and late exponential phase (**D**).

The killing kinetics of compound **11** (Figure 1A–D) were performed in two types of experiments on approximately 5 × 10^5^ (exponential phase) and 5 × 10^7^ (late exponential phase) colony forming units (CFU). The former was equivalent to MIC setup and equal to an optical density at 600 nm (OD_600_ ~ 0.0005). Colistin and chloramphenicol was used as control antibiotics against *P. aeruginosa* and *S. aureus*, respectively. Chloramphenicol was chosen against *S. aureus* because initial experiments indicated **11** to be bacteriostatic.

**Figure 2:**
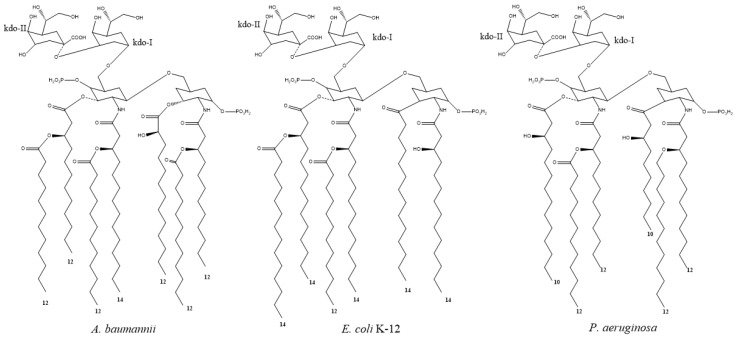
Lipid A structure of (**A**) *A. Baumannii*, (**B**) *E. coli*, and (**C**) *K. pneumonia. E. coli* and *K. pneumonia* primarily contain hexa-acetylated Lipid A, while *A. baumannii* share much similarity but lipid A is primarily hepta-acetylated. *P. aeruginosa* differs from the other Gram-negative species in that, its hexa-acetylated Lipid A had a different distribution of Acyl chains and the acyl chains were generally shorter. Numbers below acyl chains indicated the length of the carbon chain. Figures are adapted from Raetz [37], Boll [38], Li [39], and Kim [40].

A study by Bulitta et al. describes that colistin displays a rapid bactericidal activity against *P. aeruginosa* at low CFU/ mL but at higher CFU/mL, a higher colistin concentration was required to achieve activity [35]. Therefore, we decided to do time-kill experiments against late exponential phase cells (OD_600_ ~0.1 = 10^7^ CFU) to determine if **11** had the same cell density dependency as most antimicrobial peptides [36]. Against *P. aeruginosa* in exponential phase, both compound **11** and colistin were bactericidal at different concentrations tested (Figure 1A). At 1× MIC of compound **11** we observed a ~2-log reduction in viable bacterial counts and at 3–5× MIC of **11** resulted in *a* >3 log reduction in CFU within the first hour. For 1–3xMIC concentrations of **11**, the reduction in CFU was followed by regrowth, after the first hour of treatment. Against *P. aeruginosa* in late exponential phase (Figure 2B), both **11** and colistin had virtually no effect at 1× MIC, while we observed a 1-log reduction in CFU for **11** at 3× MIC and 5× MIC for colistin. The largest effect was seen for **11** at 5× MIC, where we observed a 3-log reduction in CFU. For all concentrations, regrowth occurred after 1 h.

As experiments were performed at cell densities comparable to MIC, determination of killing kinetics indicated that our MIC measurements could be slightly underestimated since regrowth was seen at 1× MIC of compound **11** to comparable levels as untreated control sample. However, treatment with 1× MIC of colistin showed the same trend, therefore we believe that these differences are due to differences in physiological conditions in time-kill assays compared to MIC: time-kill assays were performed in blood glass tubes with vigorous agitation and optimal growth condition, whereas MICs were performed in small volumes in microtiter plates where aeration is less optimal. 

For compound **11** at 1× MIC, 3× MIC, and 5× MIC against *S. aureus* in the exponential phase (Figure 1C), we observed a 2-log and >3-log reduction, respectively. Compound **11** exhibited a partially concentration-dependent killing profile, in which above 3× MIC no further reduction in CFU was observed. Killing appeared to be maximized at 1 h after treatment. However, regrowth was observed after 24 h. Against *S. aureus* in the late exponential phase, only **11** at 5× MIC had a significant effect, where a 3-log reduction in CFU was observed, again with regrowth after 24 h. 

Overall, we observed a bactericidal activity of **11** against both *P. aeruginosa* and *S. aureus*, but with a transient activity that did not last. This might be due to physiological changes in the bacteria, such as enzymatic degradation of the compound, but we expected the major reason for this to be sequestration of the compound by dead cells and cell debris. This might be observed by dissolution of the pelleted debris and LC/MS analysis.

Importantly, we find that compound **11** had activity against *P. aeruginosa* and *S. aureus*, but no activity against *E. coli* and *A. baumannii* in MIC assays. Therefore, the activity was not directed towards bacterial structures associated with the typical Gram-negative (outer membrane), as *P. aeruginosa* shared much more similarity with *E. coli* than with *S. aureus*. This could imply that either the peptide did not disrupt the membrane or that membrane disruption was more dependent on specific structures shared by *P. aeruginosa* and *S. aureus*. We speculated that the difference in **11** activity between Gram-negative species related to differences in the membrane anchoring part of LPS, known as Lipid A. *E. coli* K-12 species and *K. pneumoniae* were described to predominantly contain hexa-acetylated Lipid A that was phosphorylated at the 1- and 4′ positions. *A. baumannii* had a similar lipid A structure, but with an extra acyl chain (hepta-acetylated) [13]. However, *P. aeruginosa* was different from *E. coli*, *K. pneumonia*, and *A. baumannii*. In *P. aeruginosa*, Lipid A was hexa-acetylated, similar to that of *E. coli* but the acyl chains were distributed differently (Figure 2). Furthermore *P. aeruginosa* Lipid A generally contain shorter acyl chains than that of the other species. Therefore, Gram-negative inter species differences might result from the acylation pattern on Lipid A or the length of acyl chains on Lipid A. However, these speculation remains to be experimentally verified. It was further speculated that the peptide was generally attracted to the bacterial surface through electrostatic interactions, as both Gram-positive and Gram-negative species have negatively charged membranes, however, the antimicrobial activity would depend on aforementioned differences in membrane composition. Therefore, it was also speculated that the peptide would generally show activity against Gram-positive species, which was unlike the nonapeptide described elsewhere [18,41], which had little antimicrobial activity by itself, but could sensitize bacteria towards other antibiotics.

## 4. Materials and Methods 

### 4.1. Materials

Chemistry: Disposable 5-mL polypropylene reactors fitted with a PTFE filter were acquired from Thermo Scientific TentaGel^®^ S RAM resin, trifluoroacetic acid (TFA), piperidine, and Fmoc-protected L-amino acids were purchased from Iris-Biotech GmbH. Disopropylamine (DIEA), Triisopropylamine (TIS), Phosphate-buffered saline (PBS), melitin, and 4-(4,6Dimethoxy-1,3,5-triazin-2-yl)-4-methylmorpholiniumtetrafluoroborate (DMTMM.BF4) were purchased from Sigma-Aldrich. COMU ((1-Cyano-2-ethoxy-2oxoethylidenaminooxy)dimethylaminomorpholino-carbenium hexafluorophosphate) and Oxyma (Ethyl (hydroxyimino)cyanoacetate), HOAt (1-Hydroxy-7-azabenzotriazole), and HATU(1-[Bis(dimethylamino)methylene]-1H-1,2,3-triazolo[4,5-b]pyridinium-3-oxid hexafluorophosphate, N-[(Dimethylamino)-1H-1,2,3-triazolo-[4,5-b]pyridin-1-ylmethylene]-N-methylmethanaminium hexa-fluorophosphate N-oxide) were purchased from GL Biochem Shanghai. DMF (Dimethylformamide, synthesis grade), DCM (Dichloromethane, optical grade), and MeCN (Acetonitrile, optical grade) were obtained from VWR. 

Peptides were purified by preparative Reverse Phase Analytical High Performance Liquid Chromatography (RP–HPLC) on a preparative WatersTM XBridgeTM BEH130 C_18_, HPLC column (5 µm 10 × 250 mm) equipped with a WatersTM Cartridge Holder PKG (10 × 10 mm). 

MicroflexTM [Bruker Corporation] FlexControl software [Bruker Daltonik GmbH] was used to obtain the spectra and the data were processed using flexAnalysis [Bruker Daltonik GmbH]. All reagents and solvents were used without further purification. 

Microbiology and Haemolysis: Non-cation adjusted Mueller–Hinton broth (MHB) media (BD BBLTM Beef extract powder, BD BactoTMCasamino acids, and DIFCO soluble starch were obtained from the Becton Dickinson and Company©, Franklin Lakes, NJ, USA. MHB media supplemented with 0.2% BSA was obtained from SigmaAldrich Co. and 0.01% acetic acid from VWR^®^. 96-well plates cell culture cluster, round-bottom, polypropylene plates polystyrene, flat-bottomed 96-well ELISA plate and V-shaped 96-well polypropylene plate were purchased from Corning^®^ Inc., Costar^®^, Corning, NY, USA. Microseal^®^ film was obtained from Bio-Rad Laboratories, Inc., Hercules, CA, USA. VersaMaxTM Tunable Microplate Reader (Molecular Devices LLC, Sunnyvale, CA, USA) and the data were evaluated on Softmax^®^ Pro (Molecular Devices LLC).

### 4.2. Peptide Synthesis

Solid-phase peptide synthesis: Peptide analogues were synthesized in 4 stages, S1, S2, S3, and S4. Peptides were synthesized on a TentaGel^®^ S RAM (90 µm) resin, with a loading of 0.22 mmol/g. The resin was weighed out into a disposable 5-mL polypropylene reactor, fitted with a PTFE filter and swelled in DMF for a minimum of two hours or overnight, and washed with DMF (3×), DCM (3×), and DMF (5×). The base-labile Fmoc group was removed by treatment with 4 mL 20% piperidine/DMF for four minutes, and reiterated three times with a DMF wash in between each treatment. After the third deprotection, the resin was washed with DMF (3×), DCM (3×), and DMF (5×). For BSI-9 and analogues **1**–**4**, the amino acids were coupled successively and a single amino acid coupling used 5 equivalents of amino acid, COMU, and Oxyma each, and 2 equivalents DIEA. Fmoc-protected amino acids were dissolved in DMF at a concentration of 0.6 M. COMU and Oxyma were weighed out per coupling and dissolved in fresh DMF at a 0.6 M concentration, just prior to coupling. The amino acid solution, COMU/Oxyma solution, and DIEA were combined and immediately transferred to the syringe, covered in tin foil and put on a shaker for 1.5–2 h. For analogues **5**–**18**, HATU, HOAt, and DIEA were used as coupling reagents, as described above. The coupling solution was discharged and the resin was washed with DMF (3×), DCM (3×), and DMF (5×). The Fmoc group was removed with 4 mL 20% piperidine/DMF for 3 × 4 min, as described here earlier, and washed with DMF (3×), DCM (3×), and DMF (5×). The next amino acid was then coupled. 

### 4.3. Peptide Macrocyclization

The Fmoc group of the last amino acid was removed (4 mL 20% piperidine, 3 × 4 min). Next, the Dmb group was removed from the Asp by treatment with 3 mL 1.5% TFA/ DCM solution, 6 times for 5 min. The resin was washed with DMF, followed by DCM and finally ethanol (EtOH). The resin was freeze-dried and transferred to a reactor fitted with a PTFE filter and swelled in DMF for 2 h.

Three equivalent DMTMM·BF_4_ was dissolved in minimum DMF, 6 equivalents of DIEA were added, and the reaction was allowed to proceed for 8 h or overnight. The resin was washed with DMF (5×). Afterwards, 3 equivalents of DMTMM·BF_4_ and 3 equivalents of DIEA were added and the reaction was left for 8 h or overnight. The resin was washed with DMF (3×), DCM (3×), and EtOH (5×). Cleavage of a small amount of resin was done to confirm sequence completion and cyclization. The resin was placed in the freeze-dryer until dry.

### 4.4. Peptide Cleavage

Cleavage was performed on half or all of the resin with a TFA:H_2_O:TIS (95:2.5:2.5) solution for 2 h. The cleavage solution was collected and concentrated with a gentle stream of N_2_, down to about 300 µL. The peptide was precipitated and washed with 4 mL cold (−20 °C) diethyl ether, 3 times. The residual ether was left overnight to evaporate. Next, the crude product was dissolved in 90% water and 10% ACN and freeze-dried, ready for analysis and purification. Peptides were identified using Matrix Assisted Laser Desorption Ionization time of flight mass spectroscopy (MALDI–ToF MS). To this end, 1 µL peptide solution and 1 µL α-cyano4-hydroxycinnamic acid matrix (10 mg/ mL in ACN:H_2_O:TFA, 50:47.5:2.5) were spotted onto a target plate, followed by detection using a MicroflexTM. FlexControl software was used to obtain the spectra and the data were processed using flexAnalysis [Bruker Daltonik GmbH]. RP–HPLC was used to assess peptide purity. The system consisted of a WatersTM In-Line Degasser AF, a WatersTM 600 pump, a WatersTM 2996 Photodiode Array Detector, and a WatersTM Symmetry C18, 4.6 × 250 mm, 5 mm column. The data were processed using Empower3 software. Purity was ≥95% for all tested peptide. Peptide purification was done by dissolving the peptides in minimal H_2_O/can, and 300–500 µL was injected into the HPLC system to achieve separation. 

### 4.5. Minimum Inhibitory Concentration Determination 

Bacterial strains (*E. coli* ATCC 29522, *S. aureus* ATCC 29213, *A. baumannii* ATCC 19606, and *P. aeruginosa* ATCC 27853) were grown overnight on LB agar plates at 37 °C. Overnight cultures were prepared by inoculation of two to three colonies in 10 mL non-cation adjusted Mueller–Hinton broth (MHB) media (BD BBLTM Beef extract powder, BD BactoTMCasamino acids, and DIFCO soluble starch). Bacterial inoculum for MIC testing was prepared as balanced cultures, as previously described [25]. The bacterial suspension was adjusted using MHB to an OD600 of 0.1, approximately 1·10^8^ colony forming units (CFU/mL), and then diluted 1:200 in MHB, to give a final bacterial suspension of 5·10^5^ CFU/mL, as described by Wiegand et al. [42]. MIC plates were prepared by dissolving peptides and gentamycin samples at concentrations of 640 µM and 320 µM, respectively, in MHB media supplemented with 0.2% BSA and 0.01% acetic acid. For MIC determination, all peptides were measured in triplicates using the protocol described by Wiegand et al. [42] for “antimicrobial peptides that require the presence of acetic acid/BSA”. In brief, 20 µL peptide solution and gentamycin solution was transferred to column 1 of 96-well plates. Next, 10 µL 0.2% BSA; 0.01% acetic acid MHB media was transferred to column 2–11. Subsequently, peptides were serially diluted two-fold in the MIC plate and 90 µL bacterial suspension was transferred to all wells in column 1–11. Next, 100 µL bacterial suspension was transferred to column 12 D–F (growth control) and 100 µL media to column 12 A–C (sterility control). After an overnight incubation at 37 °C of the MIC plates, MIC values were determined as the lowest concentration at which there was no visible bacterial growth. For the inoculum control, 10 µL of solution from a positive-growth control well from each bacterial strain was dissolved in 990 µL phosphate-buffered saline (PBS) (1:100 dilution) in an Eppendorf tube. This solution was vortexed and 100 µL transferred to another Eppendorf tube containing 900 µL PBS (1:10 dilution) and vortexed. Then, 100 µL of each dilution was spread onto the MHB media plates, in triplicates, to achieve a CFU dilution of 1:1000 and 1:10,000, respectively. The plates were incubated overnight at 37 °C. The colonies were counted and 50 colonies were expected on the 1:10,000 plates and 500 on the 1:1000 plates.

### 4.6. Hemolysis

A total of 150 µL of 5 µM melittin was added to the positive control wells 1-6H of a V-shaped 96-well polypropylene plate and left overnight. The melittin solution was removed and the positive control wells were washed 3 times with 150 µL PBS. Afterwards, a 2.5 µM melittin solution was added to the positive control wells. For the assay, 0-negative blood in EDTA was used. Blood was washed by mixing 3 mL of PBS with 1 mL of whole blood, and centrifuged for 8 min at 3000 rpm. The supernatant was discarded and 4 mL of PBS added, mixed gently, and centrifuged. This wash cycle was repeated but the last centrifuge velocity was raised to 4000 rpm. Next, 40 µL red blood cells (RBC) was suspended in 8 mL of PBS to achieve an RBC suspension of 0.5% volume/volume (v/v). Peptide solutions were prepared at 600 µM, two times the highest concentration. A total of 150 µL peptide solution was transferred to row A. Next, 75 µL PBS was transferred to wells B1 to G12, as well as H7-12 (negative control), and the peptides were serially diluted two-fold. Then, 75 µL RBC suspension was added to all wells and the plate was covered with a Microseal^®^ film and incubated for 1 h at 37 °C. Next, the plate was centrifuged for 10 min at 4000 rpm. Subsequently, 60 µL supernatant was transferred into the corresponding wells of a clear, polystyrene, flat-bottomed 96-well ELISA plate. The absorbance was read at λ = 414 nm with a VersaMaxTM Tunable Microplate Reader and the data were evaluated on Softmax^®^ Pro. The results were normalized with respect to the average positive (100%) and negative (0%) controls, as described previously [43].

### 4.7. Time-Kill Kinetics

Time-kill experiments were performed on balanced exponentially growing cultures of *S. aureus* ATCC 29213 or *P. aeruginosa* ATCC 27853. We used balanced cultures, as described in Oddo et al. [44]. In brief, overnight cultures were prepared in MHB broth at 37 °C, with shaking. Overnight cultures, were back diluted and grown exponentially for no less than 8–10 generations, before experimentation. When experiments were done at OD600 = 0.0005 ~5 × 10^5^, the cultures were back diluted into fresh MHB, heated to 37 °C from an exponentially growing culture. In the late exponential phase, cultures were simply kept in the exponential phase, until OD600 = 0.1, before experimentation.

## 5. Conclusions

The present study described the successful synthesis of 18 analogues of a cyclic peptide with a flexible linker. The analogues were synthesized in four stages, focusing on cyclization point, hydrophobicity, cationic side-chain length, and combinations of the last two. We found that compound **11** showed activity against *P. aeruginosa* and *S. aureus*, but showed no activity against *E. coli* and *A. baumannii* in MIC assays. The most active analogue **11**, had a bactericidal activity against *P. aeruginosa* and bacteriostatic activity against *S. aureus*, as determined in exponential and late exponential phase time-kill experiments. At 5× MIC, **11** was just as effective as colistin in killing *P. aeruginosa*. For *S. aureus*, **11** was significantly better than chloramphenicol at 5× MIC. The results from this study might be helpful in the design of novel cyclic antimicrobial peptides. We are currently pursuing the synthesis of fatty acid analogues of **11** to mimic known cyclic lipopeptide antibiotics such as colistin and daptomycin.

## Figures and Tables

**Table 1:** Minimum inhibitory concentration (µg/mL) and percent hemolytic activity against red blood cells.

ID		1	2	3	4	5	6	7	8	9	SA ^[a]^	PA ^[b]^	EC ^[c]^	AB ^[d]^	%H ^[e]^
BSI-9		c(Lys	Nal	Lys	Lys	Bip	O2Oc	Nal	Lys	Asn) ^[f]^	32	16	64	32	33
**Stage 1**	1	c(Lys	Nal	Lys	Lys	Bip	O2Oc	Nal	**Asn**	**Lys)**	>64	32	>64	32	ND
	2	c(Lys	Nal	Lys	**Asn**	Bip	O2Oc	Nal	Lys	**Lys)**	>64	64	>64	>64	ND
	3	c(Lys	Nal	**Asn**	Lys	Bip	O2Oc	Nal	Lys	**Lys)**	64	64	>64	64	ND
	4	c(**Asn**	Nal	Lys	Lys	Bip	O2Oc	Nal	Lys	**Lys)**	>64	64	>64	64	ND
**Stage 2**	5	c(Lys	**Phe**	Lys	Lys	Bip	O2Oc	Nal	Lys	Asn)	>64	32	>64	>64	4
	6	c(Lys	Nal	Lys	Lys	**Phe**	O2Oc	Nal	Lys	Asn)	>64	32	>64	>64	6
	7	c(Lys	Nal	Lys	Lys	Bip	O2Oc	**Phe**	Lys	Asn)	>64	64	>64	64	7
	8	c(Lys	**Phe**	Lys	Lys	**Phe**	O2Oc	**Phe**	Lys	Asn)	>64	>64	>64	>64	3
	9	c(**Dab**	Nal	**Dab**	**Dab**	Bip	O2Oc	Nal	**Dab**	Asn)	8	4	>64	32	76
**Stage 3**	10	c(**Arg**	Nal	**Dab**	**Dab**	Bip	O2Oc	Nal	**Dab**	Asn)	2	2	32	32	66
	11	c(**Dab**	Nal	**Arg**	**Dab**	Bip	O2Oc	Nal	**Dab**	Asn)	8	4	32	32	36
	12	c(**Dab**	Nal	**Dab**	**Arg**	Bip	O2Oc	Nal	**Dab**	Asn)	1	4	32	32	98
	13	c(**Dab**	Nal	**Dab**	**Dab**	Bip	O2Oc	Nal	**Arg**	Asn)	1	4	32	64	80
	14	c(**Arg**	Nal	**Arg**	**Arg**	Bip	O2Oc	Nal	**Arg**	Asn)	32	32	>64	>64	68
**Stage 4**	15	c(**Arg**	Nal	**Arg**	**Dap**	Bip	O2Oc	Nal	**Dab**	Asn)	8	16	64	>64	100
	16	c(**Arg**	Nal	**Arg**	Dab	**Phe**	O2Oc	Nal	**Dab**	Asn)	32	16	64	64	16
	17	c(**Arg**	Nal	**Dab**	Dab	**Phe**	O2Oc	Nal	**Dab**	Asn)	64	16	64	>64	3
	18	c(**Dab**	Nal	**Arg**	Dab	**Phe**	O2Oc	Nal	**Dab**	Asn)	64	16	64	>64	30

^[a]^ *S. aureus* ATCC 29213 (SA), ^[b]^ *P. aeruginosa* ATCC 27853 (PA), ^[c]^ *E. coli* ATCC 29522 (EC), ^[d]^ *A. baumannii* ATCC 19606 (AB), and ^[e]^ percent hemolytic activity against red blood cells at 150 µM. To obtain the concentration in µg/mL, multiply the molecular mass of the compound (Table S1) with 150 µM and divide by 1000, ^[f]^: Non-standard amino acids—Bip: L-biphenylalanine; Dab: L-2,4-diaminobutyric acid; Nal: 3-(2-Naphthyl)-L-alanine; and O_2_Oc: 8-amino-3,6-dioxaoctanoic acid. Residues in bold are substitutions relative to BSI-9.

## Supplementary Materials

The following are available online at https://www.mdpi.com/2079-6382/9/7/366/s1. Peptide structures, Analytical HPLC traces, and MALDI-TOF-MS spectra. Table S1. Peptide mass, and HPLC retention time and purity.

## Abbreviations

ACN	Acetonitrile
Bip	L-biphenylalanine
COMU	1-Cyano-2-ethoxy-2oxoethylidenaminooxy)-dimethylaminomorpholino-carbenium hexa-fluorophosphate
Dab	L-2,4-diaminobutyric acid
DCM	Dichloromethane
DIEA	Disopropylamine
DMF	Dimethylformamide, synthesis grade
(DMTMM·BF4)	4-(4,6-dimethoxy-1,3,5-triazin-2-yl)-4-methylmorpholiniumtetrafluoroborate
EUCAST	European Committee on Antimicrobial Susceptibility Testing
Fmoc	9-fluorenylmethoxycarbonyl
HATU	1-[Bis(dimethylamino)methylene]-1H-1,2,3-triazolo[4,5-b]pyridinium-3-oxid hexafluoro-phosphate, N-[(Dimethylamino)-1H-1,2,3-triazolo-[4,5-b] pyridin-1-ylmethylene]-N-methylmethan-aminium hexa-fluorophosphate N-oxide
HOAt	1-Hydroxy-7-azabenzotriazole
Nal	3-(2-Naphthyl)-L-alanine
O_2_Oc	8-amino-3,6-dioxaoctanoic acid
Oxyma	Ethyl (hydroxyimino)cyanoacetate
PBS	Phosphate-buffered saline
RAM	Rink amide Linker
RP-HPLC	Reverse Phase Analytical High Performance Liquid Chromatography
TFA	trifluoroacetic acid
TIS	Triisopropylamine

## References

[1] Rahbarnia L., Farajnia S., Naghili B., Ahmadzadeh V., Veisi K., Baghban R., Toraby S. (2019). Current trends in targeted therapy for drug-resistant infections. Appl. Microbiol. Biotechnol..

[2] Konaklieva M.I. (2018). Addressing Antimicrobial Resistance through New Medicinal and Synthetic Chemistry Strategies. SLAS Discov..

[3] Mulani M.S., Kamble E.E., Kumkar S.N., Tawre M.S., Pardesi K.R. (2019). Emerging Strategies to Combat ESKAPE Pathogens in the Era of Antimicrobial Resistance: A Review. Front. Microbiol..

[4] Butler M.S., Paterson D.L. (2020). Antibiotics in the clinical pipeline in October 2019. J. Antibiot..

[5] Mookherjee N., Anderson M.A., Haagsman H.P., Davidson D.J. (2020). Antimicrobial host defence peptides: Functions and clinical potential. Nat. Rev. Drug Discov..

[6] Zasloff M. (2002). Antimicrobial peptides of multicellular organisms. Nature.

[7] Brogden K. (2005). Antimicrobial peptides: Pore formers or metabolic inhibitors in bacteria?. Nat. Rev. Microbiol..

[8] Greber K.E., Dawgul M. (2017). Antimicrobial Peptides Under Clinical Trials. Curr. Top. Med. Chem..

[9] Falagas M.E., Kasiakou S.K., Saravolatz L.D. (2005). Colistin: The Revival of Polymyxins for the Management of Multidrug-Resistant Gram-Negative Bacterial Infections. Clin. Infect. Dis..

[10] Humphries R.M., Pollett S., Sakoulas G. (2013). A Current Perspective on Daptomycin for the Clinical Microbiologist. Clin. Microbiol. Rev..

[11] Liu Y.-Y., Wang Y., Walsh T.R., Yi L.-X., Zhang R., Spencer J., Doi Y., Tian G., Dong B., Huang X. (2016). Emergence of plasmid-mediated colistin resistance mechanism MCR-1 in animals and human beings in China: A microbiological and molecular biological study. Lancet Infect. Dis..

[12] Friedman L., Alder J.D., Silverman J.A. (2006). Genetic changes that correlate with reduced susceptibility to daptomycin in Staphylococcus aureus. Antimicrob. Agents Chemother..

[13] Trent M.S., Ribeiro A.A., Doerrler W.T., Lin S., Cotter R.J., Raetz C.R.H. (2001). Accumulation of a Polyisoprene-linked Amino Sugar in Polymyxin-resistant Salmonella typhimurium and Escherichia coli: Structural Characterization and Transfer to Lipid A in the Periplasm. J. Biol. Chem..

[14] Band V.I., Weiss D.S. (2015). Mechanisms of Antimicrobial Peptide Resistance in Gram-Negative Bacteria. Antibiotics.

[15] Tran T.T., Munita J.M., Arias C.A. (2015). Mechanisms of drug resistance: Daptomycin resistance. Ann. N. Y. Acad. Sci..

[16] Chen C.H., Lu T.K. (2020). Development and Challenges of Antimicrobial Peptides for Therapeutic Applications. Antibiotics.

[17] Koo H.B., Seo J. (2019). Antimicrobial peptides under clinical investigation. Pept. Sci..

[18] Vaara M. (2019). Polymyxin Derivatives that Sensitize Gram-Negative Bacteria to Other Antibiotics. Molecules.

[19] Molchanova N., Hansen P.R., Franzyk H. (2017). Advances in Development of Antimicrobial Peptidomimetics as Potential Drugs. Molecules.

[20] Oddo A., Thomsen T.T., Britt H.M., Løbner-Olesen A., Thulstrup P.W., Sanderson J.M., Hansen P.R. (2016). Modulation of Backbone Flexibility for Effective Dissociation of Antibacterial and Hemolytic Activity in Cyclic Peptides. ACS Med. Chem. Lett..

[21] Ong Z.Y., Wiradharma N., Yang Y.Y. (2014). Strategies employed in the design and optimization of synthetic antimicrobial peptide amphiphiles with enhanced therapeutic potentials. Adv. Drug Deliv. Rev..

[22] Staubitz P., Peschel A., Nieuwenhuizen W.F., Otto M., Götz F., Jung G., Jack R.W. (2001). Structure-function relationships in the tryptophan-rich, antimicrobial peptide indolicidin. J. Pept. Sci..

[23] Bluhm M.E.C., Knappe D., Hoffmann R. (2015). Structure-activity relationship study using peptide arrays to optimize Api137 for an increased antimicrobial activity against Pseudomonas aeruginosa. Eur. J. Med. Chem..

[24] Ifrah D., Doisy X., Ryge T., Hansen P. (2005). Structure-activity relationship study of anoplin. J. Pept. Sci..

[25] Zhong C., Zhu N., Zhu Y., Liu T., Gou S., Xie J., Yao J., Ni J. (2020). Antimicrobial peptides conjugated with fatty acids on the side chain of D-amino acid promises antimicrobial potency against multidrug-resistant bacteria. Eur. J. Pharm. Sci..

[26] Velkov T., Roberts K.D., Nation R.L., Wang J., Thompson P.E., Li J. (2014). Teaching ‘Old’ Polymyxins New Tricks: New-Generation Lipopeptides Targeting Gram-Negative ‘Superbugs’. ACS Chem. Biol..

[27] Kondejewski L.H., Lee D.L., Jelokhani-Niaraki M., Farmer S.W., Hancock R.E.W., Hodges R.S. (2002). Optimization of Microbial Specificity in Cyclic Peptides by Modulation of Hydrophobicity within a Defined Structural Framework. J. Biol. Chem..

[28] Oddo A., Münzker L., Hansen P.R. (2015). Peptide Macrocycles Featuring a Backbone Secondary Amine: A Convenient Strategy for the Synthesis of Lipidated Cyclic and Bicyclic Peptides on Solid Support. Org. Lett..

[29] Oddo A., Nyberg N.T., Frimodt-Moller N., Thulstrup P.W., Hansen P.R. (2015). The effect of glycine replacement with flexible omega-amino acids on the antimicrobial and haemolytic activity of an amphipathic cyclic heptapeptide. Eur. J. Med. Chem..

[30] Tossi A., Sandri L., Giangaspero A. (2000). Amphipatic, a-helical anticrobial peptides. Pept. Sci..

[31] Uggerhøj L.E., Poulsen T.J., Munk J.K., Fredborg M., Sondergaard T.E., Frimodt-Moller N., Hansen P.R., Wimmer R. (2015). Rational Design of Alpha-Helical Antimicrobial Peptides: Do’s and Don’ts. ChemBioChem.

[32] Almaaytah A., Qaoud M.T., Khalil Mohammed G., Abualhaijaa A., Knappe D., Hoffmann R., Al-Balas Q. (2018). Antimicrobial and Antibiofilm Activity of UP-5, an Ultrashort Antimicrobial Peptide Designed Using Only Arginine and Biphenylalanine. Pharmaceuticals.

[33] McCoy L.S., Roberts K.D., Nation R.L., Thompson P.E., Velkov T., Li J., Tor Y. (2013). Polymyxins and Analogues Bind to Ribosomal RNA and Interfere with Eukaryotic Translation in Vitro. ChemBioChem.

[34] Blondelle S.E., Simpkins L.R., Pérez-Payá E., Houghten R.A. (1993). Influence of tryptophan residues on melittin’s hemolytic activity. Biochim. Biophys. Acta (BBA).

[35] Bulitta J.B., Yang J.C., Yohonn L., Ly N.S., Brown S.V., Hondt R.E., Jusko W.J., Forrest A., Tsuji B.T. (2010). Attenuation of Colistin Bactericidal Activity by High Inoculum of Pseudomonas Aeruginosa Characterized by a New Mechanism-Based Population Pharmacodynamic Model. Antimicrob. Agents Chemother..

[36] Savini F., Luca V., Bocedi A., Massoud R., Park Y., Mangoni M.L., Stella L. (2017). Cell-Density Dependence of Host-Defense Peptide Activity and Selectivity in the Presence of Host Cells. ACS Chem. Biol..

[37] Raetz C.R.H., Reynolds C.M., Trent M.S., Bishop R.E. (2007). Lipid A Modification Systems in Gram-Negative Bacteria. Annu. Rev. Biochem..

[38] Boll J.M., Tucker A.T., Klein D.R., Beltran A.M., Brodbelt J.S., Davies B.W., Trent M.S. (2015). Reinforcing Lipid A Acylation on the Cell Surface of *Acinetobacter baumannii* Promotes Cationic Antimicrobial Peptide Resistance and Desiccation Survival. mBio.

[39] Li Y., Yun J., Liu L., Li Y., Wang X. (2016). Identification of Two Genes Encoding for the Late Acyltransferases of Lipid A in Klebsiella pneumoniae. Curr. Microbiol..

[40] Kim S., Patel D.S., Park S., Slusky J., Klauda J.B., Widmalm G., Im W. (2016). Bilayer Properties of Lipid A from Various Gram-Negative Bacteria. Biophys. J..

[41] Ofek I., Cohen S., Rahmani R., Kabha K., Tamarkin D., Herzig Y., Rubinstein E. (1994). Antibacterial synergism of polymyxin B nonapeptide and hydrophobic antibiotics in experimental gram-negative infections in mice. Antimicrob. Agents Chemother..

[42] Wiegand I., Hilpert K., Hancock R.E.W. (2008). Agar and broth dilution methods to determine the minimal inhibitory concentration (MIC) of antimicrobial substances. Nature.

[43] Oddo A., Hansen P.R. (2017). Hemolytic Activity of Antimicrobial Peptides. Methods Mol. Biol..

[44] Oddo A., Thomsen T.T., Kjelstrup S., Gorey C., Franzyk H., Frimodt-Møller N., Løbner-Olesen A., Hansen P.R. (2016). An all-D amphipathic undecapeptide shows promising activity against colistin-resistant strains of Acinetobacter baumannii and a dual mode of action. Antimicrob. Agents Chemother..

